# Supplementary material for: Seasonal modulation of deep slow-slip and earthquakes on the Main Himalayan Thrust
Source: Nat Commun. 2018 Oct 8;9:4140. doi: 10.1038/s41467-018-06371-2 (PMC6175945; doi:10.1038/s41467-018-06371-2)
Supplement: Supplementary file 1 — Supplementary Information [file 41467_2018_6371_MOESM1_ESM.pdf]

Supplementary information

Seasonal modulation of deep slow-slip and earthquakes on the Main Himalayan Thrust

Dibyashakti Panda[1], Bhaskar Kundu[1*], Vineet K Gahalaut[2], Roland Bürgmann[3], Birendra Jha[4], Renuhaa Asaithambi[4], Rajeev Kumar Yadav[5], Naresh Krishna Vissaa[1], Amit Kumar Bansal[6]

[1]Department of Earth and Atmospheric Sciences, NIT Rourkela, Rourkela, India
[2]National Centre for Seismology, Ministry of Earth Sciences, New Delhi, India
[3]Department of Earth and Planetary Science, University of California, Berkeley, USA
[4]Department of Chemical Engineering and Materials Science, University of Southern California, USA
[5]Department of Earth Sciences, IIT-Kanpur, India
[6]CSIR-National Geophysical Research Institute, Hyderabad, India

[*]**Corresponding author:** Bhaskar Kundu, Department of Earth and Atmospheric Sciences, NIT Rourkela, Rourkela-769008, India, (rilbhaskar@gmail.com).

Supplementary Figures:

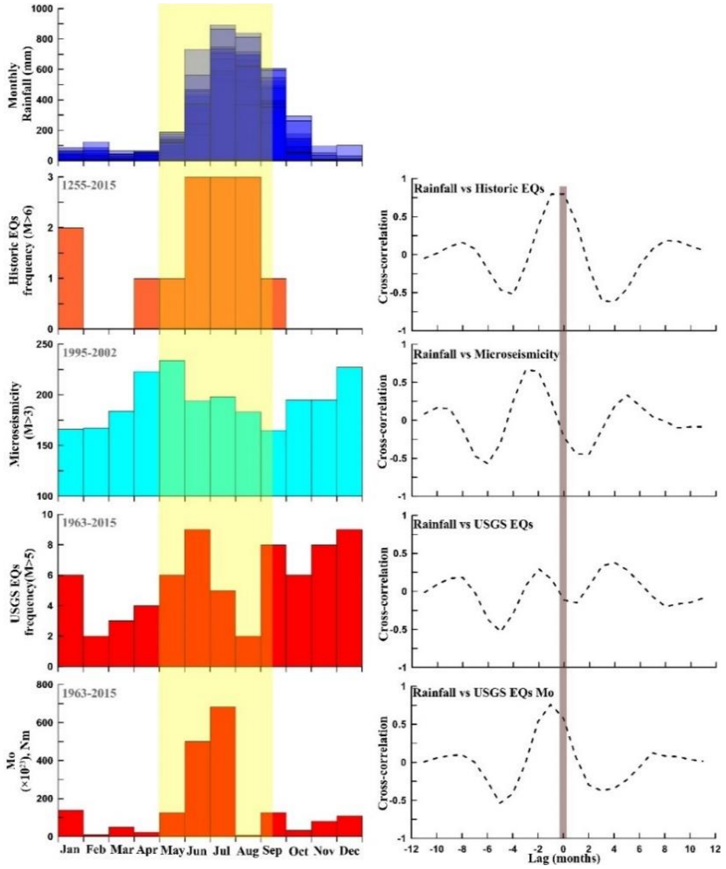

Supplementary Figure 1. *Left panel*: Stacked panels of monthly rainfall in the region surrounding Nepal, historic earthquake frequency (1255-2015 of M>6), micro-seismicity from Nepal (1995-2002 of M>3), current earthquake frequency from USGS catalog (1963-2015 of M>5) and USGS catalog seismic moment (Mo, Nm) in a region between latitude: 25°-30.5°N and Longitude: 75°-88°E. *Right panel*: Cross-correlation between rainfall and various seismic parameters. The yellow bar in the left panel marks the period of the seasonal loading deformation. Note rainfall vs historic earthquake and rainfall vs USGS seismic moment show good cross-correlation.

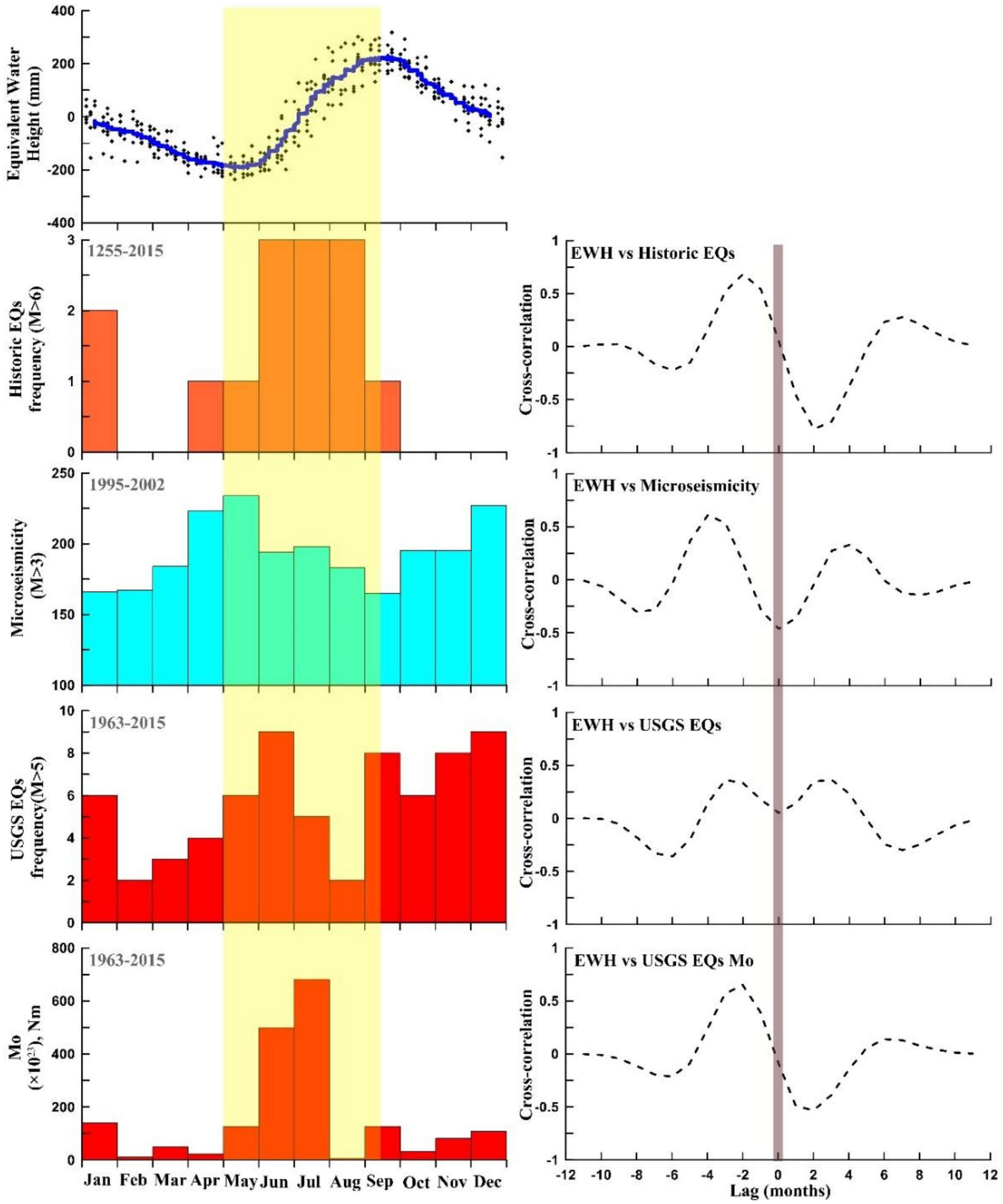

Supplementary Figure 2. *Left panel*: Stacked panels of equivalent water height (derived from GRACE averaged over the region latitude: 20°-27.5°N, longitude: 75°-88°E), historic earthquake frequency (1255-2015 of M>6), micro-seismicity from Nepal (1995-2002 of M>3), current earthquake frequency from USGS (1963-2015 of M>5) and USGS catalog seismic moment (Mo, Nm). *Right panel*: Cross-correlation between EWH and monthly seismicity number (and seismic moment from USGS current seismicity).

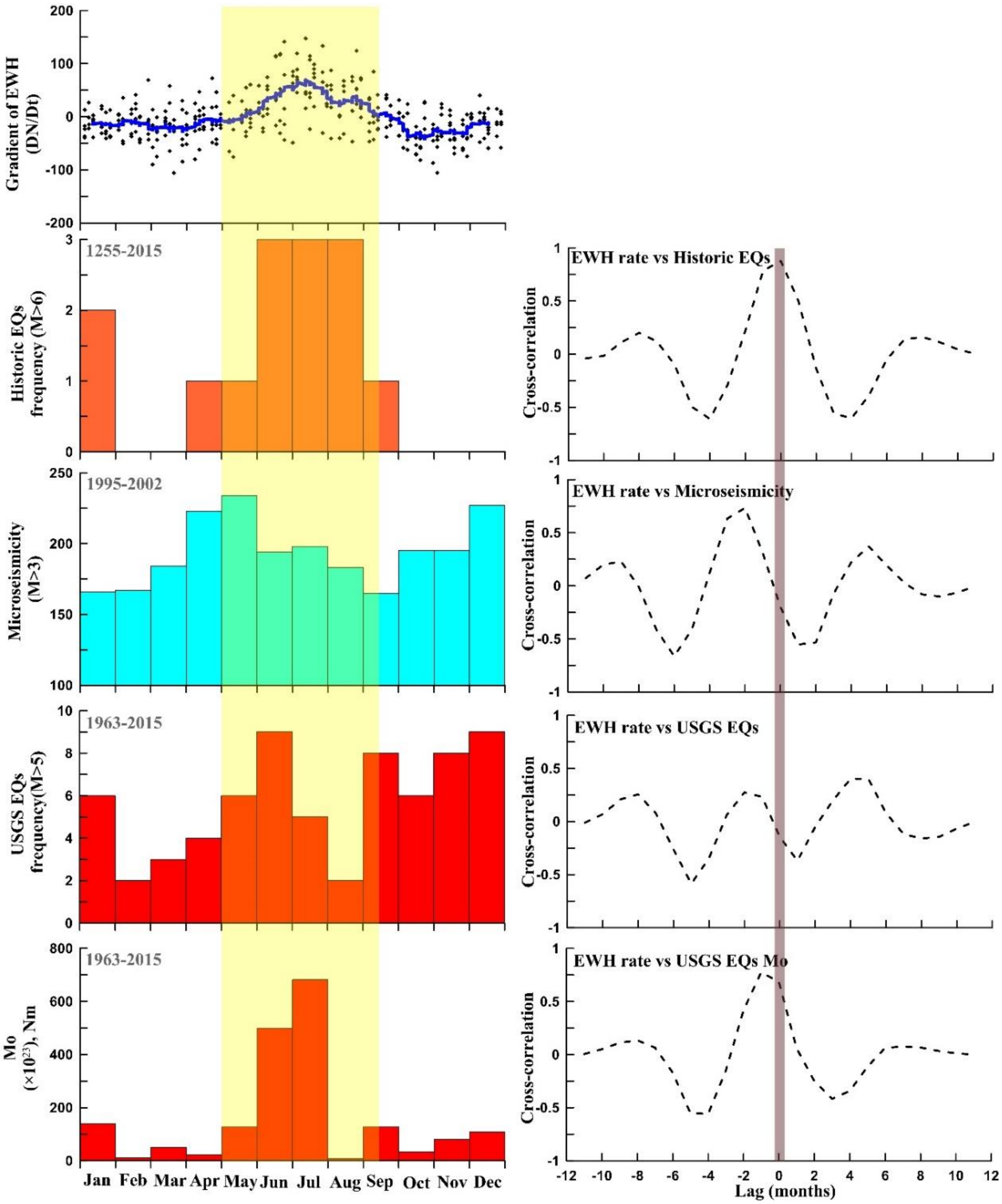

Supplementary Figure 3. *Left panel*: Stacked panels of equivalent water height gradient/rate, historic earthquake frequency (1255-2015 of M>6), micro-seismicity from Nepal (1995-2002 of M>3), current earthquake frequency from USGS (1963-2015 of M>5) and USGS catalog seismic moment (Mo, Nm). *Right panel*: Cross-correlation between EWH rate and monthly seismicity number (and seismic moment from USGS current seismicity). Note EWH rate vs historic earthquake and EWH rate vs USGS seismic moment show good cross-correlation.

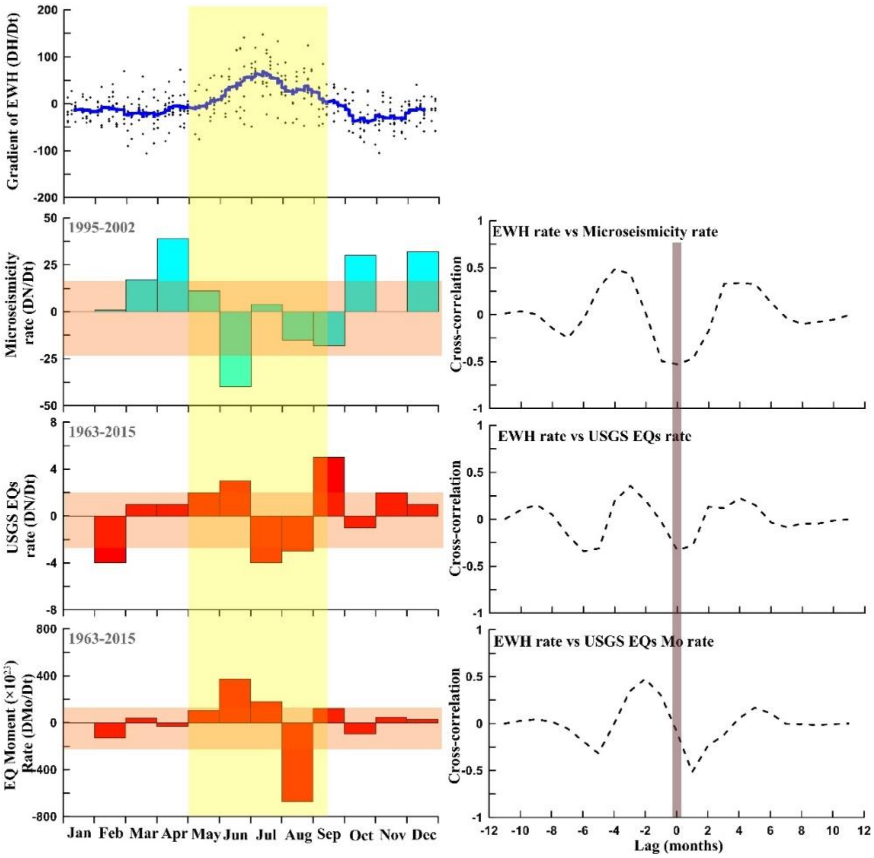

Supplementary Figure 4. *Left panel:* Stacked panels of equivalent water height gradient/rate and monthly gradient of event rates (DN/Dt) of micro-seismicity from Nepal (1995-2002 of M>3), current earthquake frequency from USGS (1963-2015 of M>5) and USGS seismic moment rate (DMo/Dt). *Right panel:* Cross-correlation between EWH rate and monthly rate of seismicity number (and seismic moment rate from USGS current seismicity).

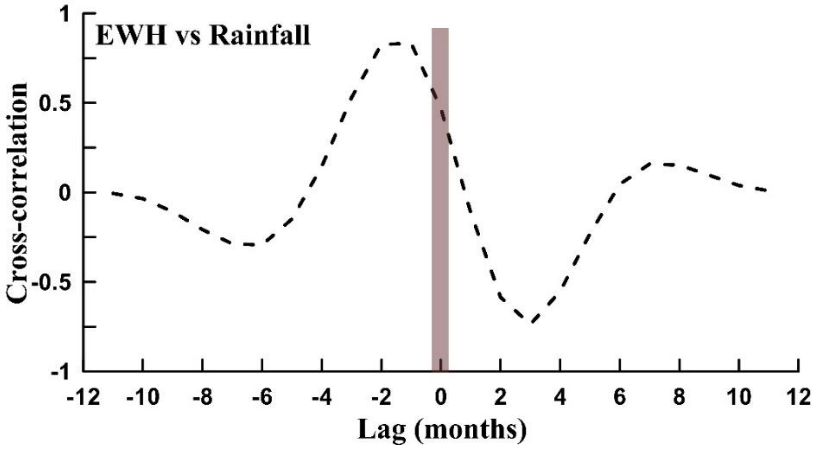

Supplementary Figure 5. Cross-correlation between GRACE derived EWH and rainfall. Note the obvious time lag of about 1.5 month (rainfall lagging EWH), with strong correlation value of ~0.75. EWH is related to the cumulative rainfall and hence its peak appears once the rainfall seasonal is over.

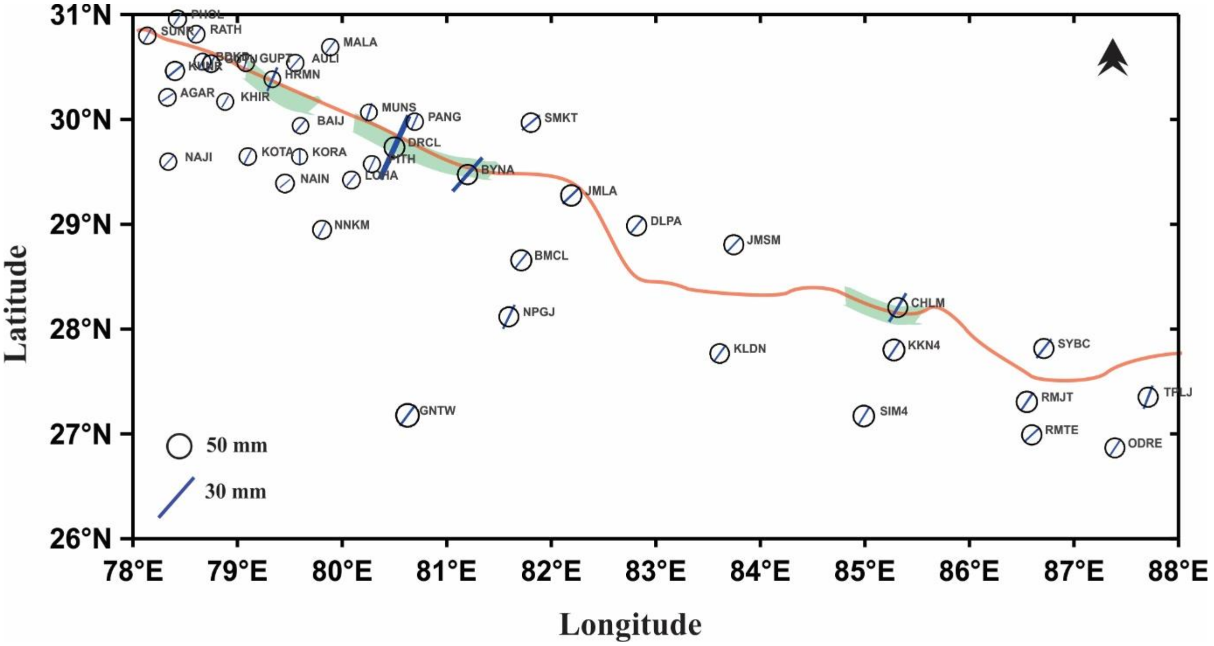

Supplementary Figure 6. Map showing spatial distribution of cGPS transients, showing the amplitude and orientation of the horizontal component (bars) and amplitude of the vertical component (black circles). Light red line marks the interseismic locked zone. Note the abnormally high transient value evident on the cGPS sites (marked by green strips) along the base of the locked zone.

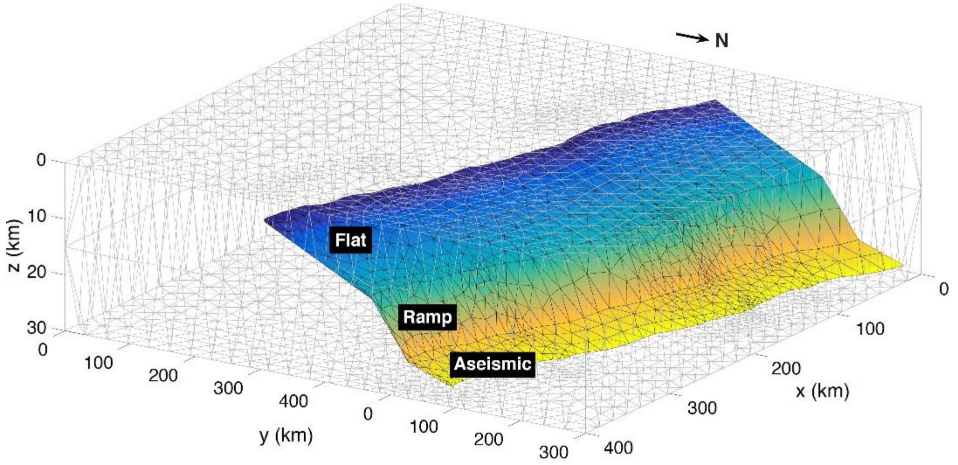

Supplementary Figure 7. The coupled flow and geo-mechanical simulation domain with the MHT fault surface and its three segments. North is aligned with the y-axis of the domain. The northward dipping MHT surface is color-coded with depth that increases from the flat segment to the aseismic segment along the fault.

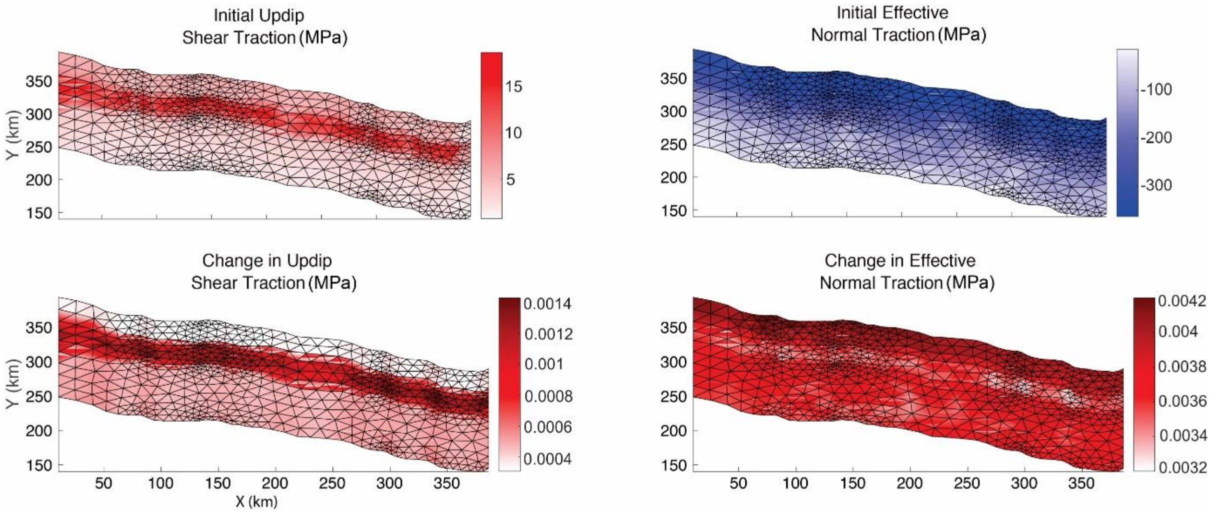

Supplementary Figure 8. *Top row:* Initial up-dip shear and effective normal traction along the MHT reflect the thrust faulting tectonic stress condition in the region. The up-dip shear traction is highest in the ramp segment due to its dip while the effective normal traction is highest in the aseismic region due to depth-dependent tectonic stresses. Negative effective normal traction indicates compression whereas positive normal traction indicates tension. *Bottom row:* The changes in up-dip shear and effective normal tractions on MHT calculated for the period of October 2004 to May 2005, which are representative of minimum and maximum hydrological load times, respectively.

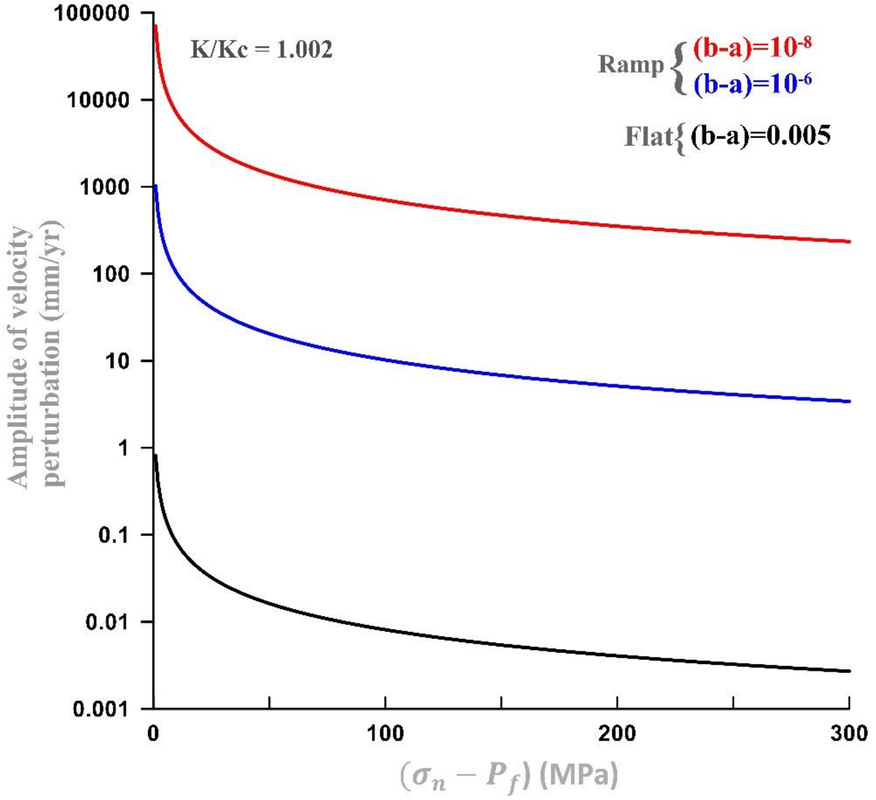

Supplementary Figure 9. Increase in amplitude of velocity perturbation (i.e., fault resonance process) with increase in pore-fluid pressure (or lowering effective normal stress).
